# Single-Step Synthesis of Vertically Aligned Carbon Nanotube Forest on Aluminium Foils

**DOI:** 10.3390/nano9111590

**Published:** 2019-11-09

**Authors:** Fabien Nassoy, Mathieu Pinault, Jérémie Descarpentries, Thomas Vignal, Philippe Banet, Pierre-Eugène Coulon, Thomas Goislard de Monsabert, Harald Hauf, Pierre-Henri Aubert, Cécile Reynaud, Martine Mayne-L’Hermite

**Affiliations:** 1 NIMBE, CEA, CNRS, Université Paris-Saclay, CEA Saclay, 91191 Gif-sur-Yvette, France; nassoy.fabien@gmail.com (F.N.); mathieu.pinault@cea.fr (M.P.); cecile.reynaud@cea.fr (C.R.); 2 NAWA Technologies, Chez STMicroelectronics, 190 avenue Célestin Coq, Cedex 13106 Rousset, France; jeremie.descarpentries@nawatechnologies.com (J.D.); thomas.goislard@nawatechnologies.com (T.G.d.M.); harald.hauf@nawatechnologies.com (H.H.); 3 Laboratoire de Physicochimie des Polymères et des Interfaces (LPPI, EA 2528), Université de Cergy-Pontoise, Cedex 95031 Neuville-sur-Oise, France; thomas.vignal1@u-cergy.fr (T.V.); philippe.banet@u-cergy.fr (P.B.); pierre-henri.aubert@u-cergy.fr (P.-H.A.); 4 LSI, CEA/DRF/IRAMIS, École Polytechnique, CNRS, Institut Polytechnique de Paris, 91128 Palaiseau, France; pierre-eugene.coulon@polytechnique.edu

**Keywords:** vertically aligned carbon nanotubes, aerosol-assisted catalytic chemical vapour deposition, Al foils, energy dispersive X-ray spectrometry, supercapacitors

## Abstract

Vertically aligned carbon nanotube (VACNT) forests are promising for supercapacitor electrodes, but their industrialisation requires a large-scale cost-effective synthesis process suitable to commercial aluminium (Al) foils, namely by operating at a low temperature (<660 °C). We show that Aerosol-Assisted Catalytic Chemical Vapour Deposition (CCVD), a single-step roll-to-roll compatible process, can be optimised to meet this industrial requirement. With ferrocene as a catalyst precursor, acetylene as a carbon source and Ar/H_2_ as a carrier gas, clean and dense forests of VACNTs of about 10 nm in diameter are obtained at 615 °C with a growth rate up to 5 µm/min. Such novel potentiality of this one-step CCVD process is at the state-of-the-art of the multi-step assisted CCVD processes. To produce thick samples, long synthesis durations are required, but growth saturation occurs that is not associated with a diffusion phenomenon of iron in aluminium substrate. Sequential syntheses show that the saturation trend fits a model of catalytic nanoparticle deactivation that can be limited by decreasing acetylene flow, thus obtaining sample thickness up to 200 µm. Cyclic voltammetry measurements on binder-free VACNT/Al electrodes show that the CNT surface is fully accessible to the ionic liquid electrolyte, even in these dense VACNT forests.

## 1. Introduction

Energy storage is a major challenge for the development of renewable energies, mobile devices and electric vehicles. In this context, supercapacitors, based on rapid ion charge/discharge cycles, have great potential, with performance between capacitors and batteries [1]. In order to develop a high-energy density, the electrode must be thick enough (a few tens of µm) and made of densely-packed active materials with a large specific surface area (SSA), a high pore connectivity and a good electronic conductivity. In commercial EDLC (Electrical Double Layer Capacitors) supercapacitors, the electrode material is usually made of activated carbon, which is a highly porous material with large SSA. However, its specific capacitance (SC) does not increase linearly with its SSA due to a too-large pore size distribution, in which the narrowest pores are not accessible to solvated ions [2]. Also, it suffers from a slow diffusion kinetics of the electrolyte in the porosities and a poor electrical contact with the current collector [3]. In order to obtain narrow pore size distributions and a better transport of charges, carbon nanomaterials are investigated [4]. Among them, carbon nanotubes (CNT) are considered thanks to their large SSA, high chemical and thermal stability, and good electrical conductivity [5,6,7]. In particular, vertically aligned carbon nanotubes (VACNTs) exhibit even better performance thanks to their anisotropy [8,9]. Moreover, binder-free VACNT-based electrodes can be prepared through deposition of conductive polymers [10] or metallic oxides [11] onto CNT surface to provide higher energy density devices [12]. Finally, the growth of VACNT onto metallic substrates is feasible [13,14,15], so that the contact resistance with the current collector is significantly reduced. Aluminium (Al) is the material industrially used as the current collector. Therefore, the direct growth of VACNT on Al foils at a relatively low cost through a scalable process must be developed to obtain competitive electrodes for supercapacitors.

The renowned method for the synthesis of high-quality VACNT is Catalytic Chemical Vapour Deposition (CCVD) [16]. In particular, aerosol-assisted CCVD [17,18] is an atmospheric pressure one-step process well suited for industrial scale-up [19,20]. In this process, the reactor is continuously fed by both carbon and catalyst precursors, involving catalyst nanoparticles formation in the gas phase [21] and subsequent deposition on substrates, where they act as seeds for the growth of multiwalled carbon nanotubes without any addition of enhancer. VACNT carpets with high thickness (up to several mm) and growth rate (up to 60 µm/min) are easily synthesized at moderate temperature (above 750 °C) [20,21]. This one-step CCVD process is robust and easier to perform than the multi-step CCVD process that requires additional steps to pre-form the catalytic particles on the substrate. In addition, the one-step process is transposable to a continuous roll-to-roll (R2R) process [15,22] which is advantageous for a low cost and efficient VACNT industrial production. However, operating such a process at a lower temperature (<660 °C), to make it suitable for the growth on aluminium substrate, needs innovative developments.

In this context, our objective is to finely tune this one-step CCVD process to grow VACNT on Al foils. To meet the requirement of industrial viability, VACNT must be clean with a thickness above 100 µm [10] and a growth rate as high as possible. However, for a direct growth on Al foils, the synthesis temperature must be below the Al melting point (*ca.* 660 °C). As the growth is thermo-activated, a drop in temperature will directly impact the growth rate. Therefore, the process must be precisely adapted in order to be efficient at such a low temperature.

In the literature, very few studies of VACNT synthesis with this single-step method are reported at a low (<650 °C) temperature [11,15]. Thickness and growth rate obtained on Al substrates are limited (less than 60 µm and 2 µm/min, respectively) and no parametric study is available. Most of the VACNT syntheses on Al substrates at a low temperature reported in the literature have been obtained through a multi-step CCVD process [23,24,25,26,27,28,29,30]. The greatest carpet thickness reported so far, with pure thermal multi-step CCVD, is about 100 µm with a growth rate about 3 µm/min [23,24]. Recently, Miura et al. [25] obtained a carpet of 1 mm with a CO_2_-assisted process, but the growth rate is limited (1.5 µm/min). With a plasma- or hot filament-assisted process, thickness of 180 µm and growth rate of 6 µm/min are reached [26,27]. However, such processes need to be operated at low pressure that is not compatible for a low-cost industrial process. To increase the maximum thickness, syntheses at longer duration were performed, but the thickness saturated after 30–60 min synthesis duration [24,25,28,29,30]. This phenomenon was explained by different mechanisms such as atomic diffusion of catalyst along the grain boundaries of aluminium [24], feedstock diffusion resistance of the growing carpet [28], or catalyst poisoning [25,29,30].

Given the state-of-the-art, it appears crucial to perform experimental studies bringing both the availability of an industrial and cost-effective one-step process for the growth of VACNT on aluminium and the understanding of their formation at such a low temperature. In the present paper, the development of the single-step aerosol-assisted CCVD synthesis of VACNTs is performed on commercial Al foils at a low temperature. A parametric study is carried out around 600 °C and consists in varying the composition of the reactive gas phase (gas ratio and flow rate) and in studying its effect on the VACNT growth as well as on their structural and electrochemical characteristics. The effect of the synthesis duration on the growth rate and on the interface between the Al substrate and the VACNT carpet is also investigated, to have a better understanding of the growth mechanisms at a low temperature. Finally, the up-scaling of the process is reported with its best performance.

## 2. Materials and Methods

VACNT are synthesized on aluminium substrates through the aerosol-assisted CCVD process, operated at atmospheric pressure in an experimental set-up (Figure S1 in Supplementary Materials) adapted from those described previously [21,31,32]. The catalytic precursor (ferrocene, 99%, Acros Organics, part of Thermofisher Scientific, Illkirch, France) dissolved in toluene (99.9%, Analytical grade, Merck, Sigma-Aldrich Chemistry products, St. Quentin Fallavier, France) is injected as an aerosol through an engine injection system (Qualiflow-Jipelec, Montpellier, France) and carried by a gas flow of argon and hydrogen into a reactor placed in a furnace set at a temperature well below the Al melting point (660 °C). Acetylene, known as the carbon precursor to be the most reactive at such a low temperature, is added to the gas phase [16]. Hydrogen is added to argon because it has been shown to have a significant impact on the decomposition kinetics of ferrocene [32] and thus, on the nucleation in the gas phase of iron nanoparticles at the origin of the formation of the catalytic particles on the Al surface. Two grades of Al are used: one provided by the SATMA PPC company, Goncelin, France (99.99% purity, thickness: 95 µm), and the other one by Fisher Scientific, part of Thermofisher Scientific, Illkirch, France (99% purity, thickness: 60 µm). Several thin Al discs (1 cm in diameter) are placed in the isothermal area of the reactor. No specific pre-treatment of the Al surface (except acetone cleaning) is required. Two synthesis temperatures are tested: 580 and 615 °C.

The thickness and morphology of the VACNT carpets are observed by Scanning Electron Microscopy (SEM, Carl Zeiss Ultra 55). The thicknesses, reported in the following results, are an average of 4 measurements taken on two cross-sections of VACNT samples always located at the same places in the centre of the reactor. CNT structure and diameter are determined by Transmission Electron Microscopy (TEM, Philips CM 12, 120 kV) after ultrasonic dispersion of CNTs in pure ethanol and deposition on lacey carbon film on copper grids. The inner and outer diameters are obtained from the observation of at least 100 CNTs per sample. Some samples are analysed by high-resolution TEM (HRTEM, JEOL 2010 F equipped with a Gatan ORIUS SC200 CCD camera) to observe the structure of CNT walls. Raman analyses (Renishaw Invia Reflex, λ_laser_ = 532 nm, P_laser_ = 2 mW) are performed from top view of the carpets. Thermogravimetric analysis (TGA, 92–16.18 SETARAM apparatus), performed under air at a heating rate of 10 °C/min up to 1000 °C, is used to determine the iron content that is calculated from the iron oxide residue obtained after full oxidation of the samples at 1000 °C.

Thin slices (10–100 nm thick) of VACNT carpet grown on Al discs are prepared with a Focused Ion Beam microscope (FIB FEI Scios) in order to perform morphology and elemental analysis of the VACNT/Al interface with scanning TEM coupled to Energy Dispersive X-Ray Spectroscopy (EDX) analysis (probe-corrected TEM FEI Titan Themis). For this purpose, a special preparation has been developed in order to homogeneously impregnate, with a resin, the inter-tube space in the VACNT carpet and the VACNT/Al interface to subsequently ensure that the sample is not altered by the preparation (see Figures S2–S4 in Supplementary Materials).

Electrochemical characterizations are performed with a multichannel potentiostat/galvanostat (VSP-150, Biologic Scientific Instrument). A three electrodes cell is used with VACNT/Al as working electrode. The electrochemical cell is home-made and consists in a stainless steel (s.s.) plate on which the VACNT/Al (working electrode (WE)) is installed in the centre. A polytetrafluoroethylene (PTFE) tube is added and screwed all together with the s.s. plate (see design on Figure S5 in Supplementary Materials). Ag wire (reference electrode (RE)) is added so that it is positioned close to the VACNTs (5 mm from the top of the VACNT forest). Then, a counter electrode (CE), made of porous carbon embedded into a s.s. grid, is added on top of the WE (VACNT) and RE electrodes. This counter electrode is composed of a mixture of activated carbon (80%), carbon black (15%) and PTFE (5%), pressed on a 316 L s.s. grid playing the role of current collector. The geometry of the three electrodes cell is thus defined with a controlled distance between WE and RE of about 5 mm. The electrolyte is a mixture of 1-ethyl-3-methylimidazolium bis(trifluoromethanesulfonyl)imide (EMITFSI, 99%, Solvionic) and acetonitrile (ACN, 99.5%, VWR International, Fontenay sous Bois, France), both used as received. The molar concentration is 1.3 M of EMITFSI in ACN. Cyclic voltammetry measurements at different scan rates (5, 10, 20, 50, 100, 200 and 500 mV/s) between −0.3 and 1.0 V are performed and used to determine the capacitance of the electrodes. The capacitance (*C* in F) of VACNT carpets, the gravimetric capacitance (*C*_m_ in F/g), as well as the developed surface area of VACNT on the Al disc (*S*_dev_ in m^2^) are determined from raw electrochemical data and from VACNT geometric data (see section “Electrochemical characterization” in Supplementary Materials).

## 3. Results and Discussion

### 3.1. Synthesis and Characteristics of Vertically Aligned Carbon Nanotube (VACNT) on Aluminium Foils

#### 3.1.1. Low-Temperature Conditions to Form Suitable VACNT Carpets

**Figure 1:**
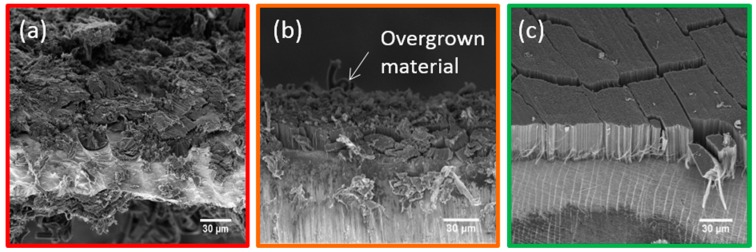
Scanning electron microscope (SEM) images of carbon nanotube (CNT) synthetized at 580 °C on thin aluminium (Al) discs during 20 min with: (**a**) high Fe/C ratio (>4 wt.%) and (**b**) medium Fe/C ratio (between 2 and 4 wt.%) and (**c**) low Fe/C ratio (<2 wt.%).

The first VACNT synthesis on thin Al discs is performed at 580 °C using similar parameters as the ones reported in previous work on quartz substrates at higher temperature with our aerosol-assisted CCVD set-up [32], using a toluene/ferrocene mixture, during 20 min. In these conditions, CNT growth is not significant: only a thin carpet (1 µm) of entangled CNT is obtained. Subsequently, acetylene is used as carbon feedstock, since it is well known that C_2_H_2_ decomposition is much easier than toluene at such a low temperature [16,25]. Keeping all the other conditions similar, VACNT cannot be observed on the Al discs (Figure 1a), stressing the importance to vary the synthesis conditions in order to improve VACNT growth on aluminium. Therefore, a first scan of the synthesis parameters, such as total gas flow rate and acetylene concentration in the gas mixture, is performed, and indicates that the Fe/C mass ratio in the precursors (defined as the mass ratio between iron in ferrocene and carbon in acetylene) has a very strong impact on the arrangement of CNT. For high Fe/C ratio (>4 wt.%), growth is definitely disrupted whatever the other parameters (Figure 1a). For medium ratio (between 2 and 4 wt.%), VACNT can be obtained but with some overgrown material at the carpet top, which is composed of bundles of CNTs (Figure 1b). Such overgrown material has already been reported in the literature, especially after long synthesis duration (>40 min) [18,28]. Lower Fe/C ratio (<2 wt.%) enables to get very clean VACNT carpets without any overgrown material (Figure 1c).

**Figure 2:**
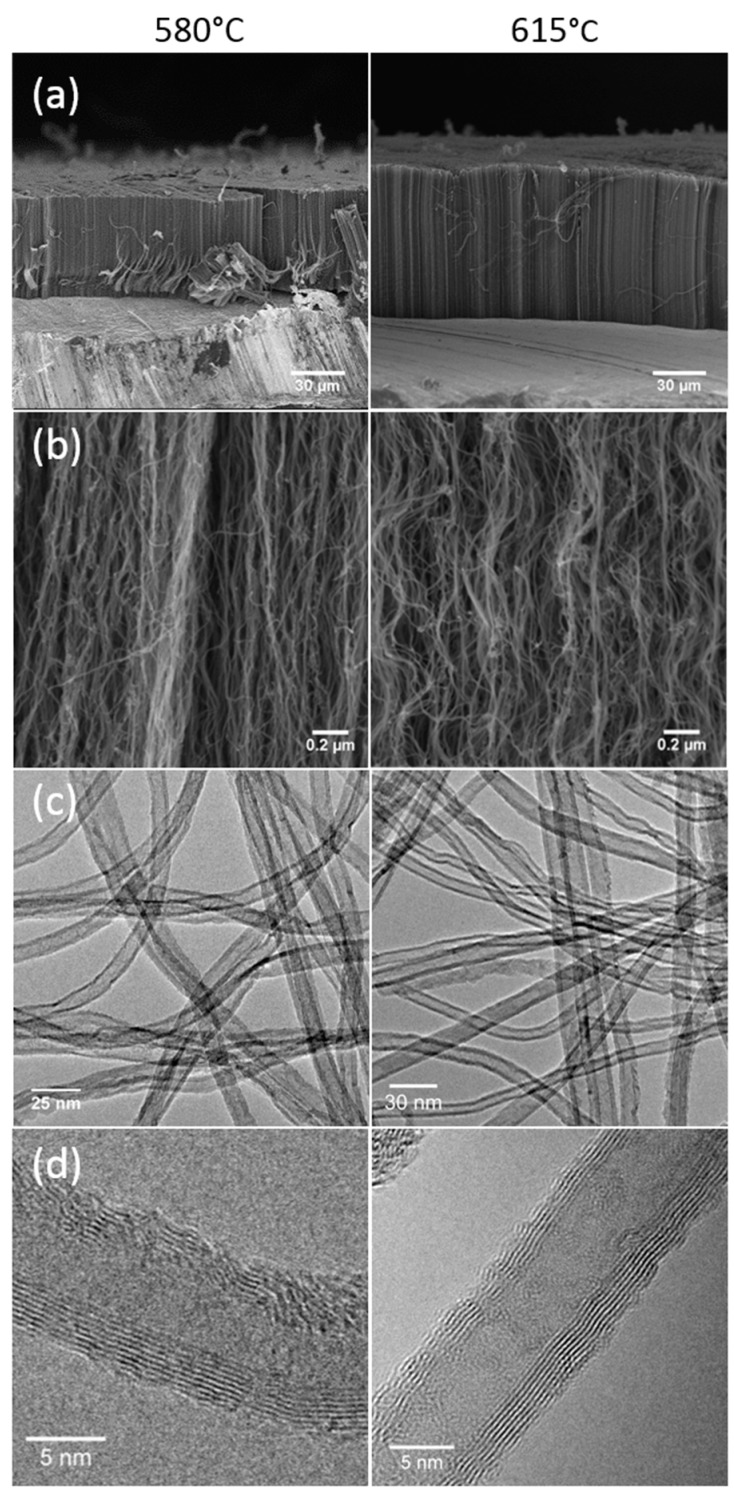
(**a**,**b**) SEM, (**c**) transmission electron microscopy (TEM) and (**d**) high-resolution TEM (HRTEM) images of VACNT obtained in optimized synthesis conditions at 580 °C (left) and 615 °C (right), respectively.

Further studies, performed at both 580 and 615 °C, show that such clean VACNT carpet can be obtained repeatedly on Al samples when the Fe/C ratio in the precursors is kept lower than 2 wt.%. The other parameters are lying in the 100–333 sccm/min range for the total flow rate and in the 10–20% range for acetylene and hydrogen concentration in the gas mixture. The VACNT carpets obtained at both temperatures, whose SEM images are shown in Figure 2a,b, are free of overgrown material and are well aligned. Thickness of the carpet grown at 580 °C during 20 min reaches 50 µm, while at 615 °C, it is significantly higher (>80 µm) with a growth rate of 4 µm/min. Such increase is a consequence of the VACNT thermo-activated growth. All the results reported above are obtained on aluminium discs prepared from SATMA foils. Same trends are obtained on discs prepared from Fisher Scientific foils. The comparison with results reported in the literature and obtained through a one-step CCVD process [11,15] reveals that our optimized process operated at 615 °C enables to grow more rapidly VACNT on both types of aluminium foils, and is even competitive as compared to the results obtained with two-step processes [5,23,24,28,29,30].

#### 3.1.2. Morphological and Structural Characterization

TEM images (Figure 2c) show clean CNTs at both synthesis temperatures, with very few by-products, such as nanoparticles located on CNT surface or encapsulated iron-based nanowires. This is in accordance with the high C purity of CNT (99.4%) measured by Thermogravimetric Analysis (TGA) under air.

Raman analysis of VACNTs obtained in optimized conditions at 580 and 615 °C reveals the occurrence of the classical D, G, D3 and 2D bands (Figure S6 in Supplementary Materials). The intensity and bandwidth of the D-band as well as the presence of the D3 band are related to the presence of defects in the graphitic structure [33]. The ratio between the D and G bands (ID/IG), commonly used to evaluate CNT quality, is around 1.3 for both synthesis temperatures, a value similar to the one obtained at 590 °C by Singh et al. [18], meaning that the CNT structure is noticeably defective. In the case of such high ID/IG ratio, the ID/I2D ratio is considered to be more relevant to qualify the structural order [34,35]. Indeed, the ID/I2D ratio is significantly lower for VACNT samples obtained at 615 °C as compared to 580 °C (4.25 as compared to 6.94). Moreover, the width of the D-band is thinner for samples synthesised at 615 °C. Therefore, the 615 °C sample exhibits a better structural quality, in agreement with the expected role of the synthesis temperature on the structural disorder [18].

Histograms of CNT diameter distributions exhibit a nearly Gaussian-type profile and reveal a very low dispersion (Figure S7 in Supplementary Materials). At 580 °C, the average outer and inner diameters are 8.7 and 4.0 nm respectively, with a standard deviation of only ±1.6 nm and ±1.1 nm respectively, while at 615 °C, they reach 10.6 (±2.3) nm and 5.1 (±1.5) nm, respectively. Such CNT diameter distributions are highly narrow, as compared to CNT obtained at higher temperatures (800 to 850 °C) with the same process [32]. HRTEM analysis indicates that CNT are composed of 7 to 9 graphene walls (Figure 2d). Some defects are occurring in the structure, but almost no amorphous carbon is observed on the CNT surface. The CNT number density in the carpets, estimated from the measured carpet weights and CNT mean diameters and lengths, reaches around 2 10^11^ CNT/cm^2^ at 580 °C and 10^11^ CNT/cm^2^ at 615 °C. Such a density is one to two orders of magnitude higher than in VACNT samples obtained at a higher temperature with the same process [32]. This result suggests a much higher number of iron nanoparticles of much smaller size formed at a lower temperature in the gas phase with a small final size due to the low coarsening of iron particles on alumina [16]. This result will be discussed below in relation with the size of the catalyst nanoparticles as observed on the substrate.

#### 3.1.3. Electrochemical Characterization

With the aim to develop Electronic Double Layer Capacitors (EDLC) supercapacitor electrodes, samples of VACNT carpets grown on Al discs with the optimized synthesis parameters determined above (named P1 to P7) are characterized electrochemically (Table 1). By playing with the synthesis duration and temperature, a set of samples presenting different characteristics in terms of height (*H*), diameter (*D*) and CNT number density (*N*) is obtained. The most relevant quantity to characterize the samples is the total surface area developed by the nanotubes of the carpet, S_dev_, which is proportional to the three of them, *S*_dev_ = π*DHN*. Thus, in Table 1, samples are ordered according to increasing *S*_dev_ value.

**Figure 3:**
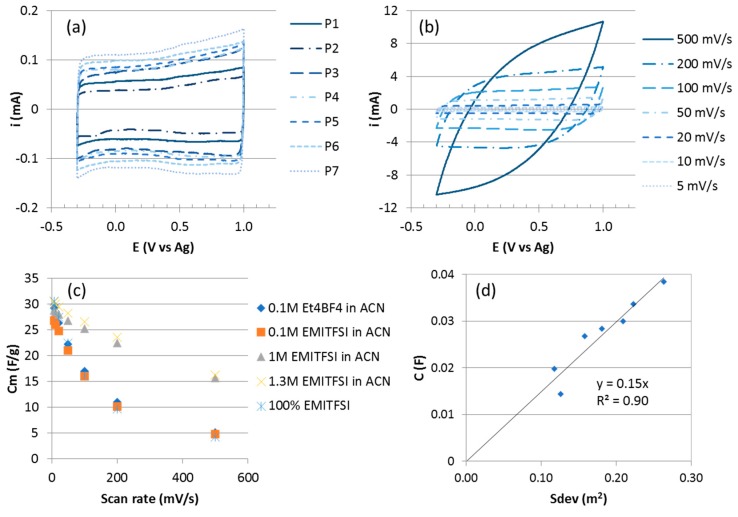
(**a**) Cyclic voltammograms (CV) curves of the different VACNT carpets in 1-ethyl-3-methylimidazolium bis(trifluoromethanesulfonyl)imide (EMITFSI) 1.3 M in ACN at 5 mV/s. (**b**) CV of P7 in EMITFSI 1.3 M in ACN at different scan rates. (**c**) Influence of the electrolyte composition on the capacitance of VACNT electrode. (**d**) Variation of capacitance with developed surface area.

Figure 3a shows the cyclic voltammetry results of electrodes P1 to P7 at a scan rate of 5 mV/s in EMITFSI/ACN at 1.3 M. The cyclic voltammograms (CV) of all the VACNT/Al electrodes show a quasi-rectangular shape characteristic of a capacitive behaviour of carbonaceous structures. The capacitance values, *C*, of all the samples are calculated from these plots and summarized in Table 1 as well as their gravimetric capacitance, *C*_m_.

For P7 exhibiting the highest capacitance, the cyclic voltammetry results at different scan rates are presented on Figure 3b. The shape remains quasi-rectangular up to 200 mV/s. At a higher scan rate, 500 mV/s, the CV exhibits a more resistive behaviour, mainly due to the electrolyte conductivity. The gravimetric capacitance decreases from 30 to 16 F/g for scan rates ranging from 5 to 500 mV/s (Figure 3c). This decrease of capacitance when the scan rate increases is found to be lower in the EMITFSI (1.3 M)/ACN medium as compared to other organic electrolytes also tested (Figure 3c). In Et_4_NBF_4_ 0.1 M in acetonitrile, Cm decreases from 29 to 5 F/g when the scan rate increases from 5 to 500 mV/s and in pure EMITFSI, Cm varies from 41 to 4 F/g.

The gravimetric capacitances (C_m_) obtained for the different samples are in the 30–47 F/g range which is included in the range reported in the literature for MWCNT (4–80 F/g) [36]. These values are higher than those of about 7 F/g obtained for VACNT carpets produced at higher temperatures (800–850 °C) on Si wafers using the same CVD process [10]. This can be explained by the higher number density and specific surface area of the VACNTs grown on aluminium (6 × 10^10^ to 2 × 10^11^ CNT/cm^2^ and 230–340 m^2^/g) as compared to the ones of VACNT on silicon substrates (10^9^ and 10^10^ CNT/cm^2^ and 50 m^2^/g). In addition, these values compare favourably with other results from the literature regarding VACNT on Al substrates. Dörfler et al. reported 32.1 to 61.2 F/g from 25 µm high VACNT carpets grown on Al foils for CNT exhibiting diameters from 9.8 to 5.6 nm [28], while Reit et al. mentioned values of 30 to 80 F/g for VACNT exhibiting heights from 5 to 7 µm [37]. However, in these studies, the specific capacitance decreases when the CNT weight increases, and rapidly falls below 40 F/g whereas in our study even for the heaviest VACNT, the specific capacitance is maintained at quite high values (see the comparison between P3 and P6 in Table 1). To explain this trend, Reit et al. mentioned the hypothesis that amorphous carbon layers, built-up during the increased duration of VACNT growth, could decrease the overall conductivity of the electrode and detrimentally impact the electric double-layer formation. In comparison to the trends reported in the literature, our results, giving rise to pure VACNT with different total surface area, emphasize the crucial role of the CNT purity and of the total surface area developed by the CNT on the electrode capacitance.

Regarding now the variation of the capacitance *C* at 5 mV/s, as a function of the developed surface area (*S*_dev_) of the VACNTs (Figure 3d), C increases quasi linearly with *S*_dev_ and the slope allows to calculate the intrinsic areal capacitance of the CNT external wall. The obtained value of 15 µF/cm^2^ is close to the one of 21 µF/cm^2^ measured for a graphene surface in similar ionic liquids [38]. The linear dependence of *C* with *S*_dev_ indicates good electrolyte accessibility to the developed surface of the VACNTs throughout the carpet in spite of its high density. It also confirms the real benefit of the anisotropic arrangement of nanotubes, thus forming porous channels favourable to electrolyte diffusion. Such results outline the benefits of the VACNT growth process on aluminium current collector described in this article that enables to get a high number density of narrow VACNTs. Such dense and anisotropic structures exhibit high potentialities as electrode materials for EDLC supercapacitors.

#### 3.1.4. Effect of Synthesis Duration on VACNT Thickness, CNT Diameter and Density

**Figure 4:**
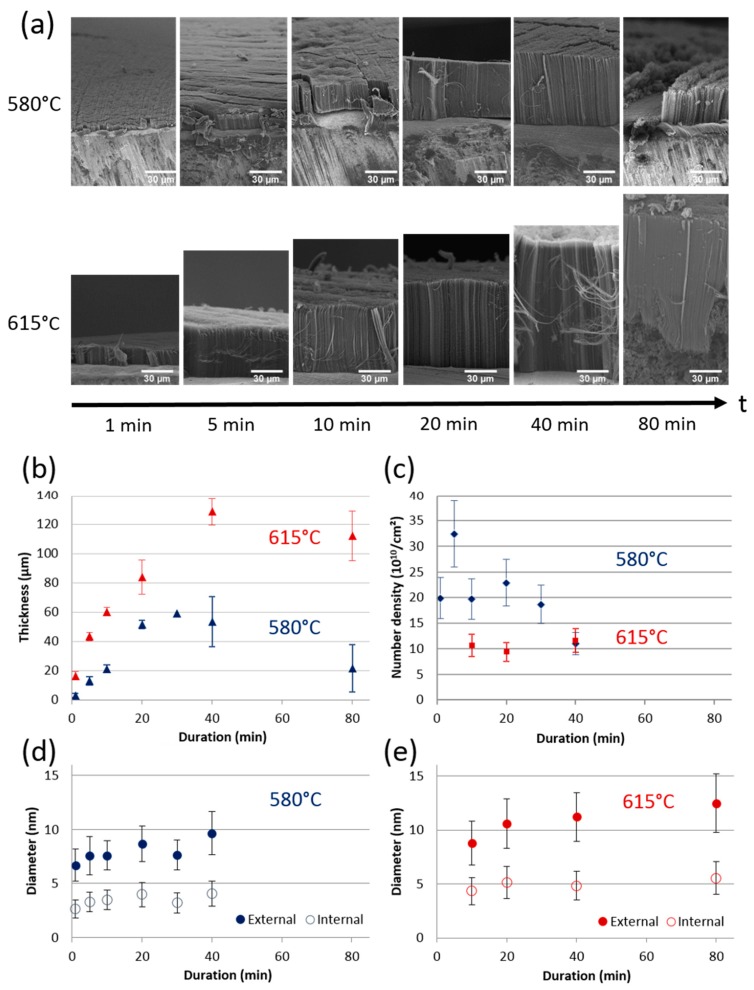
(**a**) SEM images of VACNT carpets obtained at 580 and 615 °C for increasing synthesis duration. Influence of synthesis duration on (**b**) carpet thickness, (**c**) CNT number density and (**d**,**e**) external and internal CNT diameters and their standard deviations at 580 and 615 °C, respectively.

In order to increase the developed surface area of the VACNT carpets, the carpet thickness must be increased as far as possible. The synthesis duration is thus a key parameter. Using the conditions determined above, a series of synthesis experiments, consisting in varying only the duration between 1 and 80 min, are performed at 580 and 615 °C. The corresponding SEM images of resulting samples (Figure 4a) show that well-organized carpets are formed up to 40 min synthesis duration. However, at 80 min, some disruptions along the carpet cross-sections are observed for both temperatures.

At 580 °C, the VACNT thickness varies almost linearly with the synthesis duration up to 20 min (Figure 4b), reflecting a mean growth rate about 2.5 µm/min. Then, the thickness stabilizes at 55 ± 5 µm but at 80 min it drops abruptly. Moreover, at 80 min, the CNTs cover only partially the substrate surface (Figure 4a) and only aligned CNT bundles are locally observed. At 615 °C, the thickness increases non-linearly up to 40 min of synthesis duration (with a mean growth rate of 4 µm/min), but above this duration, a saturation of thickness around 120 µm appears (Figure 4b). SEM analyses (Figure 4a) reveal that at 80 min, below the carpet, there is a significant amount of additional carbon at the VACNT/substrate interface, in which only some entangled nanotubes are distinguished instead of aligned nanotubes.

The outer diameters increase slightly as the synthesis duration increases. At 580 °C, the mean value and standard deviation vary respectively from 6.7 and 1.5 nm to 9.7 and 2.0 nm when the synthesis duration increases from 1 to 40 min, respectively (Figure 4d). At 615 °C, they vary from 8.8 and 1.3 nm to 12.5 and 1.5 nm from 10 to 80 min synthesis duration, respectively (Figure 4e). However, the mean CNT number density (Figure 4c) hardly changes with increasing duration: from the first minutes of synthesis at 615 °C, it reaches its maximum value of 10^11^ cm^−2^ and approximately twice this value at 580 °C, up to 30 min synthesis duration.

To sum up, increasing the synthesis duration above 20 min allows for getting thicker VACNT carpet only at 615 °C. 130 µm VACNT carpets are then obtained from 40 min synthesis duration. However, the variation of thickness versus synthesis duration is not linear and saturation occurs after 20 min at 580 °C and 40 min at 615 °C. It is important to note that for these durations, such trends were not observed at higher temperatures with this one-step process [31] and a linear behaviour of CNT length with synthesis duration is reported even for syntheses of several hours [39]. However, a few papers report some saturation when duration exceeds about one hour, attributed to feedstock diffusion resistance [13,18,40]. For the multi-step CCVD process, such saturation phenomena are commonly observed and are attributed to various phenomena [16]: alteration of the catalytic particles by diffusion into the substrate [24] or Ostwald ripening or lift-up [41], catalytic deactivation from the formation of carbon by-products [25,29,42] and feedstock diffusion resistance [28,43]. In the following, the different hypotheses will be examined through the analysis of the VACNTs/Al interface and a more detailed follow-up of the synthesis duration effect using sequential synthesis.

### 3.2. Understanding the Growth Rate Changes Occurring at Low Temperature

#### 3.2.1. Analysis of the Aluminium (Al)/VACNT Interface by Scanning Transmission Electron Microscopy coupled with Energy Dispersive X-Ray Spectroscopy (STEM/EDX)

**Figure 5:**
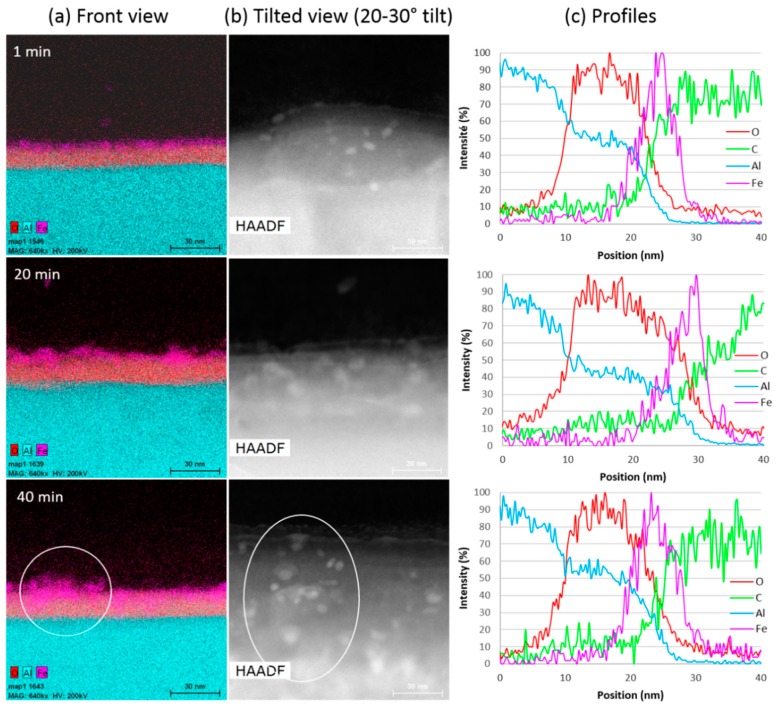
(**a**) Chemical cartography, (**b**) STEM images and (**c**) profiles of the Al/VACNT interface from syntheses at 580 °C of 1, 20 and 40 min duration, respectively (all images are at the same scale).

The decrease in growth rate observed during the VACNT synthesis by the single step CCVD process could be due to changes occurring at the interface between the Al substrate and the VACNT carpet, related in particular to the diffusion of iron in the substrate [16,24,41]. To check this hypothesis, STEM/EDX analysis of the Al/VACNT interface is performed on samples synthesized for different durations in order to get local information on morphological and chemical changes resulting from the VACNT synthesis (Figure 5, also see Figure S3 in Supplementary Materials). It is worth recalling that the substrate is a commercial Al foil without any pre-treatment. The three samples analysed are part of those presented above (Figure 4) and synthesised at 580 °C for 1, 20 and 40 min. At 580 °C, thickness saturation is total after 40 min, so this sample should provide information on the saturation origin.

The chemical analysis along the cross-section (front view, Figure 5a) allows for clearly identifying the oxide layer and the iron nanoparticles on its top. The presence of iron-based particles on the substrate surface for all samples (Figure 5a,b), as well as the observation by TEM of the carbon nanotubes attached to these particles (see Figure S3 in Supplementary Materials), is consistent with a base growth mechanism. This rules out a complete encapsulation of the catalytic particles into the inner CNT channel as the origin of the growth saturation [40]. In addition, the observed increase in the number of particles with synthesis duration shows that the iron nanoparticles resulting from the decomposition of ferrocene in the gas phase can diffuse through the growing carpet towards the substrate. The mean catalyst particle size (estimated from about ten particles in High-Angle Annular Dark-Field (HAADF) top view (Figure 5b) varies from 6 nm for 1 min duration to 8–9 nm for 20 and 40 min, which is consistent with the CNT diameters reported above (Figure 4d). This is consistent with the width of the Fe peak in the chemical profiles along the cross section of the VACNT/Al interface (Figure 5c). Thus, a slight coarsening of the catalyst particles is observed with increasing synthesis duration, as often reported in the literature [16], but only in the initial stage below 20 min synthesis duration.

The distribution of iron in the front view (Figure 4a), shows some overlaps between iron and alumina, suggesting a possible diffusion of iron in alumina for a synthesis duration of 20 min, and even more after 40 min. However, the roughness of the Al surface, which is not negligible at this scale (average roughness is close to 300 nm), can be misleading (see Figure S4 in Supplementary Materials). Actually, looking at the Al surface from above (HAADF, Figure 5b), one can see that the areas in the chemical mapping (white circle in Figure 5a,b for 40 min duration) where a high iron density is observed, correspond well to a larger density of iron particles on the surface top. In the profiles of Figure 5c, only a slight left shift of the Fe signal maximum towards the Al signal is observed when the synthesis duration reaches 40 min. Thus, there could be a slight diffusion of iron in the alumina subsurface only after 40 min of synthesis.

To sum up, STEM/EDX observations show that the catalytic nanoparticles are barely affected by iron diffusion phenomena into the subsurface and remain available on the substrate surface for CNT growth. Furthermore, their size remains approximately constant beyond 20 min, which also dismiss a stop of the growth by Ostwald ripening. Therefore, the diffusion of iron into the Al substrate is most probably not responsible for the VACNT thickness saturation.

#### 3.2.2. Sequential Synthesis

**Figure 6:**
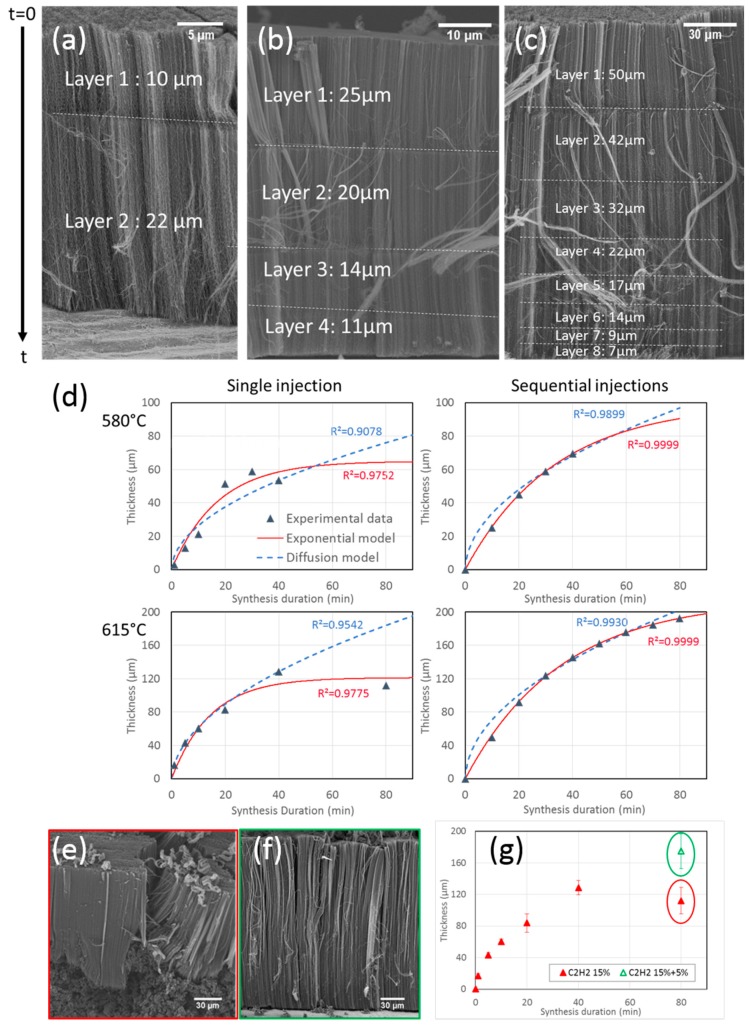
SEM images of carpets obtained with sequences of (**a**) 5 min then 10 min at 580 °C, (**b**) 4 × 10 min at 580 °C and (**c**) 8 × 10 min at 615 °C. (**d**) Fits of experimental data with exponential model (red) and diffusion model (blue). (**e**,**f**) SEM images of carpets obtained at 615 °C for 80 min without (**e**) or with (**f**) reduction of C_2_H_2_ flow rate after 20 min. (**g**) Evolution of VACNT thickness with synthesis duration without (red) or with (green) reduction of C_2_H_2_ flow rate after 20 min.

To get a better understanding of the effect of synthesis duration, sequential syntheses are undertaken. This experimental procedure consists in injections of precursors interspersed with pauses where only carrier gases are sent [31,44]. These pauses last 5 min to ensure that precursors are no longer present and thus that the reaction is stopped when the next injection is started. These pauses let a mark in the carpet so that it is possible to measure the height corresponding to the different sequence durations during the growth and therefore to access to the growth kinetics. A first experiment with a short sequence of 5 min followed by a longer one of 10 min is performed at 580 °C to verify if the base growth mechanism determined at 850 °C [31] is still in play at lower temperatures. The total injection duration of precursors is limited to 15 min, in order to avoid any VACNT growth limitation so that carpet height is proportional to injection duration. SEM observations of the VACNT carpet (Figure 6a) consist of two layers, the longest one (22 µm) is located at the bottom, on the substrate surface, and is twice longer than the top layer (10 µm), confirming the base growth mechanism.

Then, experiments with 4 sequences of 10 min performed at 580 °C, and 8 sequences of 10 min at 615 °C, are carried out. SEM images reveal 4 and 8 layers respectively, for a total thickness of 70 µm at 580 °C and almost 200 µm at 615 °C (Figure 6b,c). These total thicknesses are significantly higher than those reported above for samples prepared with the same total synthesis duration but using the standard process. In addition, it is important to note that the disordered carbon observed at the base of the carpet at 615 °C with the standard process is no longer present under the multi-layer carpet obtained with the sequential process (Figure 6c). Taking into account the base growth mechanism, the first layer to grow is at the carpet top. The layer thickness gradually decreases from the top to the bottom of the carpet, but no complete saturation is observed in contrast with the standard process trends obtained for the same total duration. Clearly, the intermediate breaks without injection of precursors allow for limiting the processes at the origin of the saturation. The growth rate decrease may be related to feedstock diffusion-limited phenomena or catalyst deactivation, as discussed below.

#### 3.2.3. Growth Rate Model

Different models have been proposed to describe the CNT length versus time in VACNT forests. Generally, the models are related either to feedstock diffusion resistance of the carpet (diffusion model (Equation (1)) [43], or to catalytic deactivation by poisoning of particles with disordered carbon. In this last case, the most used model is the exponential model (Equation (2)) which describes a self-deactivation process [42,45,46,47]:

(1) $$h\left(t\right)=1/2\left(\sqrt{A^{2}+4Bt}-A\right)$$

(2) $$h\left(t\right)=\gamma_{0}\tau \left(1-e^{-\frac{t}{\tau }}\right)=h_{max}\left(1-e^{-\frac{t}{\tau }}\right),$$

where, *h*(*t*) is the height of the carpet (e.g., CNT length). The coefficients (*A* and *B*) of the diffusion model (1) depend on the diffusion coefficient, reaction constant, carbonaceous precursor concentration on the carpet surface, and carpet density [43], (see Supplementary Materials). The coefficients of the exponential model (2) are: τ the catalyst lifetime (in min) and *γ*₀ the initial growth rate (in µm/min) [42,45,46,47].

The fittings of our experimental data with both models (Figure 6d) show that in both standard and sequential injections, the diffusion model does not correctly describe our experimental data. In standard synthesis, the model cannot account for the existence of the growth termination phenomena, while the exponential model provides a better fit. For sequential injections, the diffusion model fits the experimental data a little better, but the exponential model gives a much better result. Therefore, it seems that a phenomenon of catalytic deactivation may be at the origin of the decay of CNT growth rate when synthesis duration increases.

The synthesis temperature has a strong effect on the initial growth rates, which is enhanced by about 100% when the temperature increases from 580 to 615 °C, while the catalyst lifetimes do not vary (Table 2). However, at a given temperature, the initial growth rates are similar whatever the synthesis procedure, but the catalyst lifetimes and thicknesses of the sequential syntheses are almost twice the ones of standard syntheses.

These trends indicate that the initial growth rate is controlled by the temperature, as expected from a thermo-activated process, while the catalyst lifetime is very sensitive to the synthesis procedure. It seems that intermediate breaks in the precursor’s delivery can reactivate catalytic particles. The same interpretation regarding the effect of sequential synthesis on growth was reported by Zhang et al. [47] in the case of a multi-step CCVD process. Since the final VACNT thicknesses reached are much higher than in the standard procedure, this process adjustment may be used in an industrial context to increase the maximum thickness.

The catalytic deactivation that seems to be at the origin of the growth rate limitation is generally associated in the literature with the high reactivity of C_2_H_2_ and a decrease in its concentration is often found to increase the lifetime of the catalytic particles [16,25]. To verify this claim, another sequential synthesis is performed, but with a change in the acetylene supply. This experiment is performed at 615 °C for 20 min under the same conditions as the syntheses presented in Figure 4, then for 60 min with an acetylene flow rate divided by three. SEM observations show that the reduction in acetylene flow rate prevents the formation of the disorganized carbon under the CNT mat (Figure 6e,f). The VACNT mat reaches a mean height of 180 µm, as compared to 112 µm for the 80 min standard synthesis (Figure 6g). Therefore, limiting the C_2_H_2_ concentration allows to significantly reduce the formation of disorganized carbon and consequently, to greatly increase the catalyst lifetime, which supports the catalytic deactivation as the origin of the thickness saturation.

A growth limitation by deactivation of the catalyst particles is surprising, considering that, in the single-step process, the catalyst precursor is continuously injected so that the catalyst particles should be continuously refreshed as observed at a high temperature [48]. However, at a high temperature, the carbon precursor was not acetylene but toluene. To explain why in the present case the renewal of catalytic particles is insufficient to compensate for their deactivation, the first explanation is the higher rate of formation of disorganized carbon because of the highly reactive acetylene, as suggested by the last experiment where the acetylene supply is limited. In addition, at such low synthesis temperatures, the mobility through the CNT carpets of the iron-based nanoparticles nucleated in the gas phase is lowered so that it is not sufficient to refresh the catalytic particle located at the CNT base and to subsequently impart a continuous activity of the particle for long time CNT growth. Both phenomena, e.g., formation of disorganized carbon and catalyst particle mobility, may be in competition and the refreshment of the catalyst particles is not enough to compensate for the significant formation of disorganised carbon.

### 3.3. Toward Application: Industrial Process

**Figure 7:**
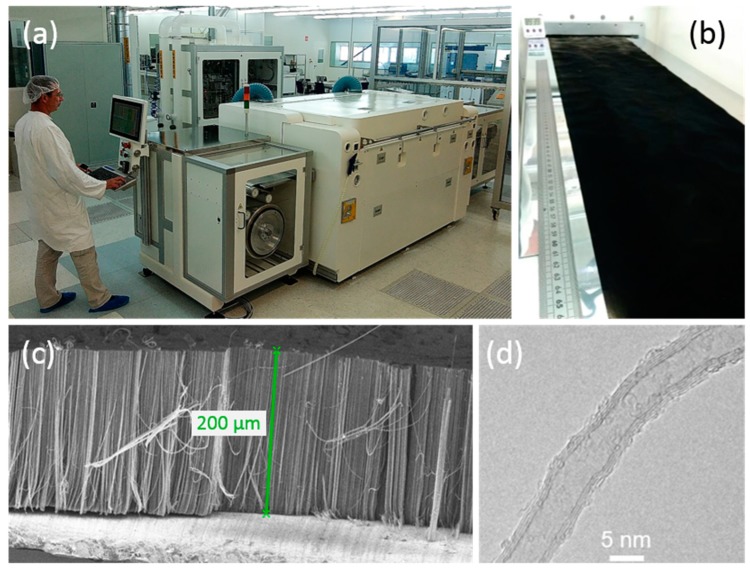
VACNT grown on a 30 cm-wide Al foil at NAWATechnologies’ roll-to-roll (R2R) production line (rolling speed of 1 m/h): (**a**) set-up, (**b**) Al foils at the oven exit, (**c**) SEM image of the grown VACNT carpet and (**d**) HRTEM image of a nanotube.

With the goal of mass producing VACNTs on Al electrodes for supercapacitors, the single-step atmospheric-pressure aerosol-assisted CCVD process, developed here at a low temperature, has been successfully scaled to a continuous 30 cm-wide roll-to-roll production tool on the NAWATechnologies’ pilot line (Figure 7a,b). The resulting carpets of VACNT can reach heights above 200 µm for 60 min growth duration at 615 °C (Figure 7c). The nanotube characteristics are very similar to those obtained on the laboratory CVD growth equipment in terms of diameter and structure, as observed by HRTEM (Figure 7d). This achievement demonstrates the transferability of the lab-scale process to an industrial scale despite the differences in terms of reactor shape, gas injection system and reactor inlet geometry, grade and thickness of the Al foil.

## 4. Conclusions

The one-step aerosol-assisted CCVD process was successfully optimized at a low temperature in order to produce VACNT forest on aluminium foils with a competitive growth rate, paving the way for its industrial development. Such novel developments are resulting from a fine control of synthesis parameters, and in particular, of the Fe/C ratio in the precursor flow. At 615 °C, clean VACNT forests of high C purity (99.5 wt.%) can be grown at a rate in the [4,5] µm/min range. It is a significant progress compared to the reported results obtained with this one-step process. Indeed, the growth rates are even competitive with those obtained with plasma or hot-filament-assisted two-step processes.

Lowering the growth temperature allows for the formation of a high number of iron-based nanoparticles of small size, so that the VACNT forest density is about 10^11^ CNT/cm^2^ at 615 °C with CNT outer diameter about 10 nm and a narrow distribution. The Raman ID/IG ratio is around 1.3 since defects are occurring in the structure, but almost no amorphous carbon is observed on the CNT surface by HRTEM. Electrochemical characterization in ionic liquids of these VACNT on Al foils, constituting binder-free electrodes, reveals a good accessibility of the nanotube surface to ions in spite of the high CNT density of the forest. Such a behaviour confirms the real benefit of the anisotropic arrangement of nanotubes to prepare performant supercapacitor electrodes.

Some saturation of the growth rate is observed for long synthesis durations, which was not observed at higher temperatures with this one-step CCVD process. STEM/EDX observations enable us to locate catalytic nanoparticles at the Al/VACNTs interface and to show that they remain available on the substrate surface for CNT growth even after long synthesis duration, which dismiss a stop of the growth by iron diffusion in Al substrate. Through the approach of sequential synthesis, we have been able to model the VACNT height saturation with an exponential decay characteristic of a self-deactivation phenomenon and to show that limiting the C_2_H_2_ concentration in the precursor flow allows for greatly increasing the catalyst lifetime. Therefore, the occurrence at a low temperature of a growth rate limitation is probably due to the propensity of C_2_H_2_ to form disorganized carbon. Such a phenomenon involves a limitation in the refreshment of the catalytic particle provided by the continuous feeding of iron precursor, which is less efficient to prevent catalyst deactivation as compared to synthesis performed without C_2_H_2_ at a higher temperature.

Finally, the process has been successfully scaled-up at the industrial level in a roll-to-roll process paving the way to the mass production of innovative supercapacitors.

## Figures and Tables

**Table 1:** VACNT characteristics and capacitance of VACNT-based electrodes.

Sample	*H*eight (µm)	*M*ass (mg)	*D*iameter (nm)	*D*ensity (10^10^ CNT/cm^2^)	*S*_dev_ (m^2^)	*C*_m_ (F/g)	*C* (mF)
P1	99	0.42	8.1	5.9	0.117	47	19.7
P2	96	0.45	8.1	6.6	0.126	32	14.4
P3	49	0.57	8.3	15.7	0.158	47	26.8
P4	65	0.81	10.6	10.6	0.181	35	28.4
P5	53.5	0.79	8.7	18.2	0.209	38	30.0
P6	56	0.84	8.7	18.5	0.223	40	33.6
P7	125	1.28	11.1	7.7	0.263	30	38.4

**Table 2:** Fitted parameters *γ*_0_ (initial growth rate), *τ* (catalyst lifetime) and corresponding *h*_max_ with the exponential model.

Coefficient	*h*_max_ (µm)	*γ*₀ (µm/min)	*τ* (min)
Single injection 580 °C	65	3.8	17
Single injection 615 °C	121	8.3	15
Sequential injection 580 °C	100	3.0	33
Sequential injection 615 °C	215	6.0	36

## Supplementary Materials

The following are available online at https://www.mdpi.com/2079-4991/9/11/1590/s1. Figure S1: Scheme of aerosol-assisted CCVD device, Figure S2: FIB Preparation of thin samples, Figure S3: TEM images of the Al/VACNT interface, Figure S4: Schematic view of the sample roughness effect on the STEM images, Figure S5: Design of the home-made electrochemical cell, Figure S6: VACNT Raman spectra, Figure S7: Histograms of VACNT diameters.
