# Supplementary material for: Single-Step Synthesis of Vertically Aligned Carbon Nanotube Forest on Aluminium Foils
Source: Nanomaterials (Basel). 2019 Nov 9;9(11):1590. doi: 10.3390/nano9111590 (PMC6915653; doi:10.3390/nano9111590)
Supplement: Supplementary file 1 [file nanomaterials-09-01590-s001.pdf]

Single-Step Synthesis of Vertically Aligned Carbon Nanotube Forest on Aluminium Foils

Fabien Nassoy[1,2], Mathieu Pinault[1], Jérémie Descarpentries[2], Thomas Vignal[3], Philippe Banet[3], Pierre-Eugène Coulon[4], Thomas Goislard de Monsabert[2], Harald Hauf[2], Pierre-Henri Aubert[3], Cécile Reynaud[1], Martine Mayne-L'Hermite[1*]

[1]*NIMBE, CEA, CNRS, Université Paris-Saclay, CEA Saclay, 91191 Gif-sur-Yvette, France*

[2]*NawaTechnologies, chez STMicroelectronics, 190 avenue Célestin Coq, 13106 Rousset Cedex, France*

[3]*Laboratoire de Physicochimie des Polymères et des Interfaces (LPPI, EA 2528), Université de Cergy-Pontoise, 95031 Neuville-sur-Oise Cedex, France*

[4]*LSI, CEA/DRF/IRAMIS, École polytechnique, CNRS, Institut Polytechnique de Paris, 91128 Palaiseau, France*

Supplementary Material

1. Aerosol-assisted CCVD device

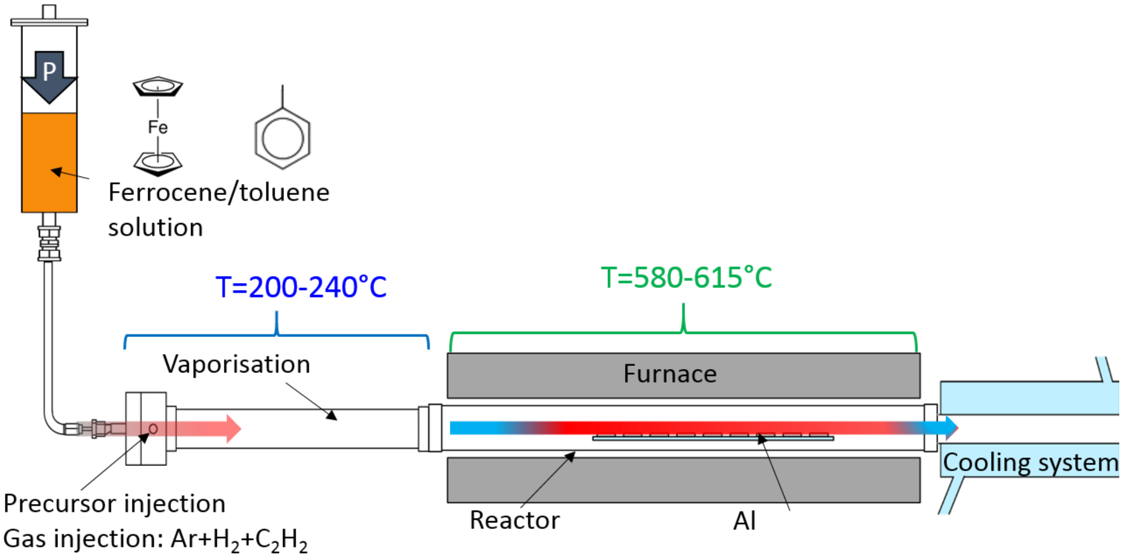

Fig.S1: Scheme of aerosol-assisted CCVD device.

2. Preparation by FIB (Focused Ion Beam) of thin samples

a) Resin filling of VACNT/Al samples

The process consists in infiltrating a solution of epoxy polymer in the intertube space [1], so that VACNTs emerge from the cutting operation without alteration. The epoxy solution is made from a commercial mixture composed of a resin (EPON, Epoxy Embedding Medium), a hardener (MNA: Methyl-5-norbornene-2,3-dicarboxylic anhydride [$C_{10}H_{10}O_3$]) and an accelerator [DMP30: (2,4,6-Tris(dimethylaminomethyl) phenol ([$(CH_3)_2NCH_2]_3C_6H_2OH$)]. The thin Al discs, covered with VACNTs, are cut and held by clips (Struers) in Teflon moulds, previously lubricated with a silicone release agent. The impregnation is performed under vacuum (CitoVac, Struers), which limits the formation of air bubbles. Two vacuum cycles are performed, then a curing treatment at 60°C for 24 hours is carried out. Finally, using an ESC 200 GTL polisher (ESCIL), the samples are gradually thinned (both in thickness and width) with polishing discs with finer and finer grains.

b) FIB cutting

The thin samples for TEM analysis are prepared from the VACNT/Al in resin composites with a FIB FEI Scios (TEMPOS equipment, NanoTEM platform, C2N Marcoussis). The different steps of the preparation are as follows:

i) The selected sampling area across the interface between Al substrate and VACNTs is identified and protected by the deposition of a platinum strip (Fig. S2a) (20 µm long, 2 µm thick), which also increases conductivity and facilitates focusing despite the insulating nature of the resin.
ii) Both sides of the platinum strip are milled with a relatively intense beam (I=3 nA), then with a less intense one (I=1 nA) near the marked area (Fig. S2b).
iii) The thin blade is cut by making a J-shaped mark ("J-cut"), then stuck on the micromanipulator with a platinum deposit (Fig. S2c & d), before finishing the J-cut so that the blade can be extracted (Fig. S2e) and glued to an appropriate TEM grid (Fig. S2f).
iv) Once the sample has been disconnected from the micromanipulator (Fig. S2g), the blade is gradually thinned (bevelled to avoid bending) with a low intensity beam so as not to damage it until it reaches a thickness of about 100 nm. The blade is then ready for TEM/EDX analysis (Fig. S2h).

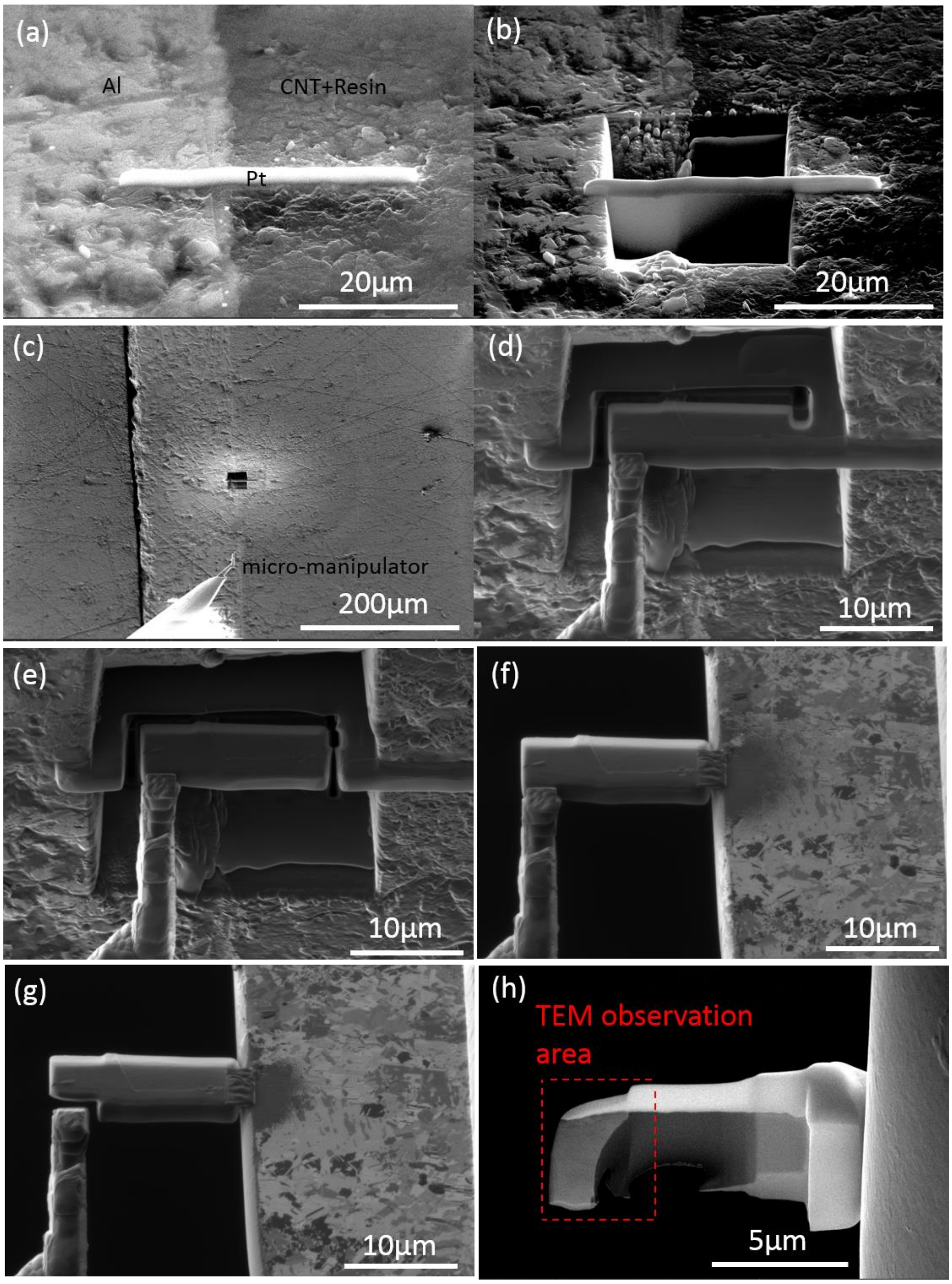

Fig.S2: Preparation by FIB of thin samples: (a) Deposit of a layer of Pt on the area to be collected; (b) Holes created by ablation of the two neighbouring areas; (c-e) Cutting and removal of the thin sample ; (f-g) Deposit of the thin sample on the special TEM grid; (h) Thinning of the sample.

c) TEM observations of the thin samples

TEM images of a thin sample, prepared from a VACNT/Al material obtained through a 20 min synthesis, are presented in Fig.S3. On the left, we can see the Pt strip deposited during the preparation by FIB, the CNTs embedded in the resin, as well as the aluminium surface. In the centre, the aligned CNTs can be distinguished, whose direction is indicated by the dotted line. Finally, on the right, the image centred on the interface reveals a layer, probably alumina, and iron-based particles on the surface of this alumina layer.

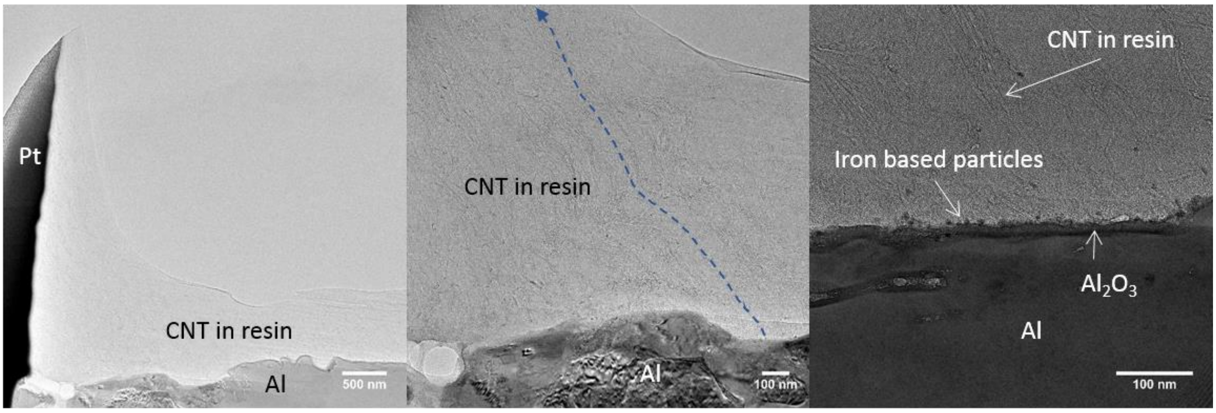

Fig.S3: TEM images of the Al/VACNT interface visible in a thin sample cut in a sample from a 20 min synthesis. The blue dotted line in the centre panel is an indication of the general direction of the CNTs embedded in the resin.

3. Conditions selected for STEM/EDX observations to take into account the sample roughness

Two viewing angles were chosen to avoid possible experimental biases that could be induced by the roughness of the sample in the EDX analysis.

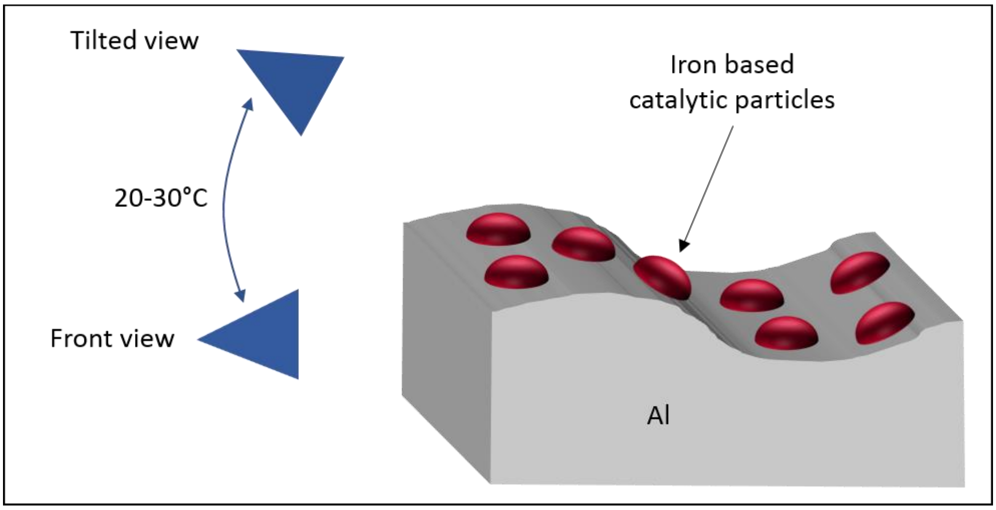

Fig.S4: Schematic view of the effect of the surface roughness of the sample on its appearance during STEM/EDX analyses and indication of the viewing angles chosen to limit its impact (note: CNT on particles are not represented).

4. Electrochemical characterisation

Design of the home-made electrochemical cell

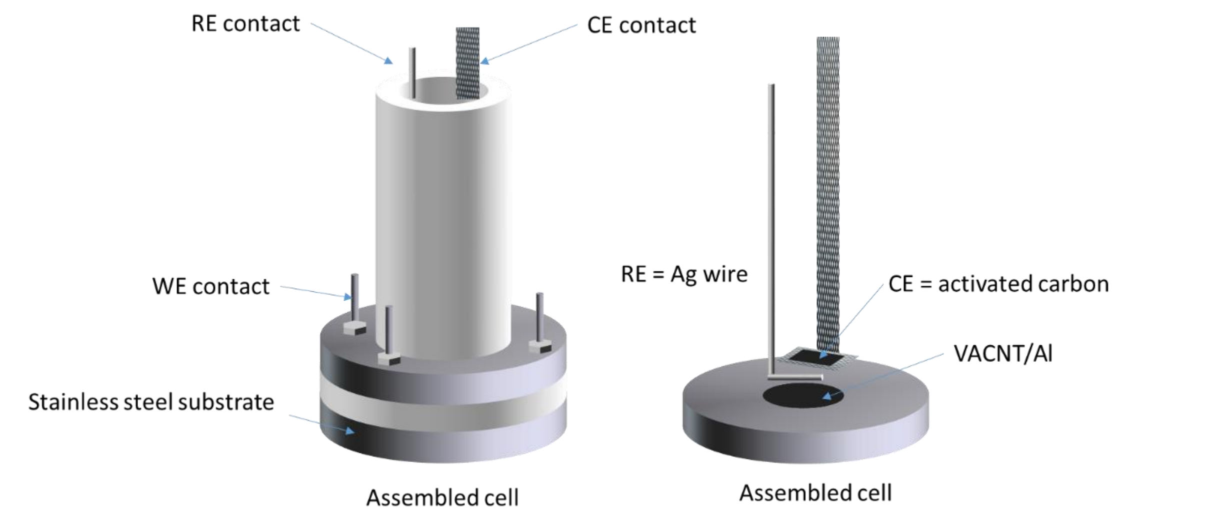

Figure S5: Design of the home-made electrochemical cell: It consists of a stainless steel (s.s) substrate on the centre of which the VACNT/Al working electrode (WE) is positioned. Then a PTFE tube is added and screwed all together with the s.s substrate. The reference electrode (RE) (Ag wire) is added so that it is positioned close to the VACNTs (5 mm from the top of the VACNT carpet). Then a counter electrode (CE) (made of porous carbon embedded into a s.s. grid) is added on top of the WE and RE electrodes. The geometry of the three-electrodes cell is thus controlled with a distance between WE and RE of about 5mm.

Determination of capacitance and developed area

The specific capacitance, C (F), of VACNT carpets is calculated using the equation:

$$C = \frac{Q_d}{\Delta V},$$

where Q_d (C) is the charge of discharge and ΔV (V) the electrochemical window. This capacitance is then used to calculate the gravimetric capacitance, C_m (F/g),

$$C_m = \frac{C}{m_{VACNT}},$$

where m_{VACNT} (g) is the weight of VACNT on the aluminium disc.
Finally, S_{dev} (m^2) is the developed area of VACNT on the aluminium disc:

$$S_{dev} = \pi.d.h.density.S,$$

where d (m) is the diameter of VACNT, h (m) its height, S the disc surface area and density (VACNT/cm^2) the CNT number density in the carpet.

5. Raman analysis of VACNTs on Al

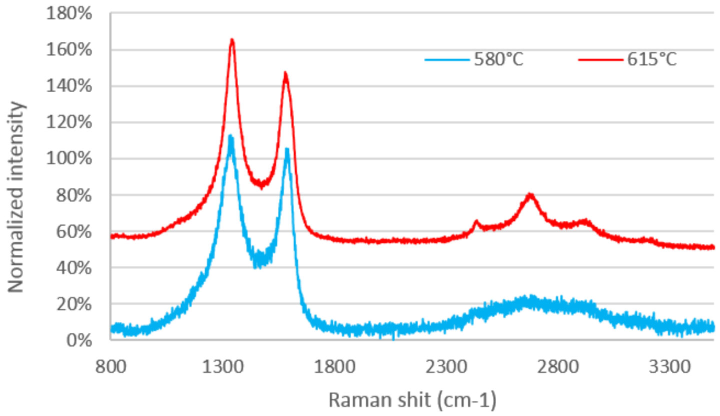

T (°C)	580	615
ID/IG	1.26	1.31
ID/I2D	6.94	4.25
ID3/IG	0.27	0.23
G (cm-1)	1582	1580
D (cm-1)	1336	1340
Width D (cm-1)	122	89

Fig.S6: Raman spectra at 580°C and 615°C (left) and relevant quantitative parameters obtained by a conventional adjustment procedure (right)

6. Histograms of diameters from TEM analysis

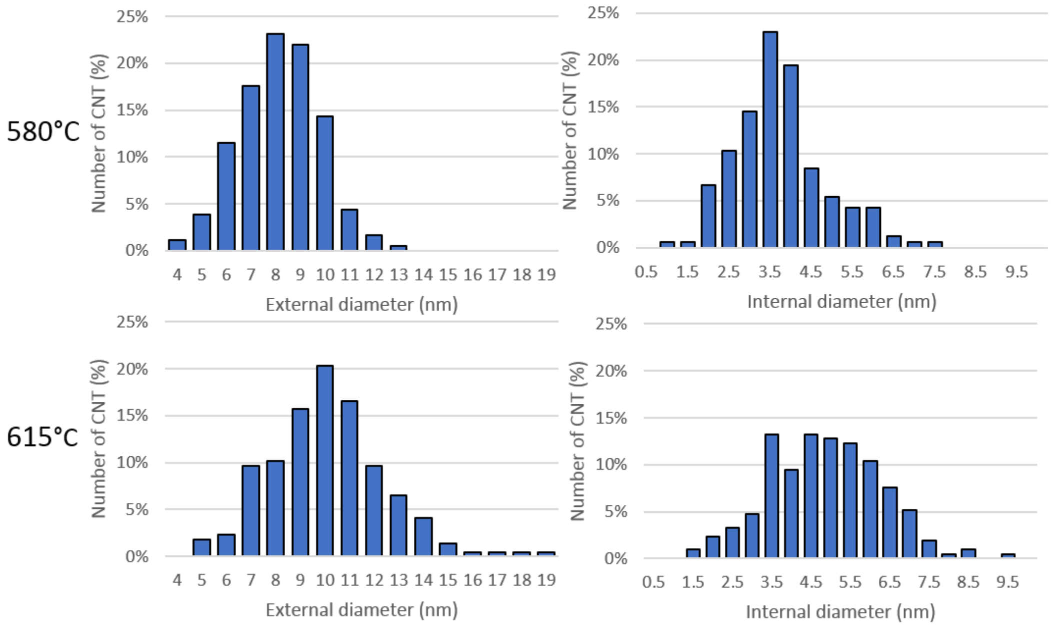

Fig.S7: Histograms of diameters: External (left) and internal (right) diameter distribution of the CNT.

7. Models of the VACNT thickness variation with synthesis duration

Diffusion model

The following model, derived from work on silicon oxidation kinetics [2] and adapted to VACNT growth by Zhu et al. [3], takes into account both reactivity and diffusion. The evolution of height $h(t)$ is given by Equation (1):

$$h(t) = 0.5\sqrt{A^2 + 4Bt} - 0.5A \qquad (1)$$

With $A = \frac{2D}{\mathrm{k}}$ and $B = \frac{4DC_0}{M}$, where D is the diffusion coefficient in the carpet (cm²/s), k is the reaction constant (cm/s), C_0 is the concentration of carbon precursor on the surface of the carpet (mol/µm³) and M is the density of the carpet (mol/µm³).

Exponential model

The exponential decay of the growth rate is often observed in the CCVD growth of VACNTs and attributed to the catalyst poisoning due to the formation of a carbonaceous layer around the catalysts particles [4–6]. The postulate is that the rate of this catalyst poisoning is proportional to the rate of the synthesis reaction and corresponds to a self-deactivation process which is expressed by Equation (2) where γ is the growth rate (in µm/min) and τ is the reaction time constant (in min).

$$\frac{d\gamma}{dt} = -\frac{1}{\tau}\gamma \qquad (2)$$

By solving this differential equation, we obtain the expression of the growth rate (3), where γ_0 is the initial growth rate.

$$\gamma(t) = \gamma_0 e^{-\frac{t}{\tau}} \qquad (3)$$

By integrating this equation with the initial condition $h(t = 0) = 0$, we obtain the evolution of the thickness as a function of time (4). The maximum achievable thickness is equal to $h_{max} = \gamma_0\tau$ (µm).

$$h(t) = \gamma_0\tau\left(1 - e^{-\frac{t}{\tau}}\right) = h_{max}\left(1 - e^{-\frac{t}{\tau}}\right) \qquad (4)$$

References

[1] Huard M, Roussel F, Rouzière S, Patel S, Pinault M, Mayne-L'Hermite M and Launois P 2014 Vertically aligned carbon nanotube-based composite: Elaboration and monitoring of the nanotubes alignment *J. Appl. Polym. Sci.* **131** n/a-n/a

[2] Deal B E and Grove A S 1965 General Relationship for the Thermal Oxidation of Silicon *J. Appl. Phys.* **36** 3770–8

[3] Zhu L, Hess D W and Wong C 2006 Monitoring carbon nanotube growth by formation of nanotube stacks and investigation of the diffusion-controlled kinetics *J. Phys. Chem. B* **110** 5445–9

[4] Einarsson E, Murakami Y, Kadowaki M and Maruyama S 2008 Growth dynamics of vertically aligned single-walled carbon nanotubes from in situ measurements *Carbon N. Y.* **46** 923–30

[5] Puretzky A A, Geohegan D B, Jesse S, Ivanov I N and Eres G 2005 In situ measurements and modeling of carbon nanotube array growth kinetics during chemical vapor deposition *Appl. Phys. A* **81** 223–40

[6] Futaba D N, Hata K, Yamada T, Mizuno K, Yumura M and Iijima S 2005 Kinetics of water-assisted single-walled carbon nanotube synthesis revealed by a time-evolution analysis *Phys. Rev. Lett.* **95** 1–4
